# Psychosocial Effects of COVID-19 in the Ecuadorian and Spanish Populations: A Cross-Cultural Study

**DOI:** 10.3389/fpsyg.2022.803290

**Published:** 2022-04-29

**Authors:** Ángela Ximena Chocho-Orellana, Paula Samper-García, Elisabeth Malonda-Vidal, Anna Llorca-Mestre, Alfredo Zarco-Alpuente, Vicenta Mestre-Escrivá

**Affiliations:** ^1^Department Educational Psychology, Faculty of Philosophy, Letters and Educational Sciences, University of Azuay, Cuenca, Ecuador; ^2^Faculty of Psychology, Department of Basic Psychology, University of Valencia, Valencia, Spain

**Keywords:** psychosocial effects, COVID-19, Ecuadorian, Spanish, cross-cultural

## Abstract

The world's population is currently overcoming one of the worst pandemics, and the psychological and social effects of this are becoming more apparent. We will present an analysis of the psychosocial effects of COVID-19: first, a cross-sectional study in an Ecuadorian sample (*n* = 301) and second, a comparative study between two samples from the Ecuadorian and Spanish populations (*n* = 83 each one). Participants completed an online survey to (1) describe how they felt (depression, anxiety, and stress) before and after confinement; (2) analyze which emotional and behavioral variables predict depressive symptoms, anxiety, and stress perceived after the confinement; (3) carry out a comparative study in a sample of Ecuadorian and Spanish surveys. Results indicate, first, that Ecuadorians experience significantly more depressive symptoms, anxiety, and stress after confinement. Second, variables which predict depressive symptoms and anxiety are greater public prosocial tendency, less stress as a challenge, and greater stress as a threat, as well as an empathetic tendency that implies greater emotional regulation. Experienced stress after confinement was predicted by a greater public prosocial tendency, as well as an empathetic tendency. Finally, scores for depression, anxiety, and stress are higher after confinement in both countries. However, results reveal the similarity of the psychosocial effects that are being experienced, regardless of the country, and the differences in the variables that can help explain these effects. This can contribute to the constitution of intervention plans which aim to soften and alleviate the effects produced by a situation such as that experienced with COVID-19.

## Introduction

The confinement due to SARS CoV-2 (COVID-19) caused the suspension of economic, scholarly, social, cultural, and political activities. This extraordinary situation has generated a great deal of biopsychosocial damage due to the loss of habits and routines that today can be seen as physical and psychological problems (Wang et al., 2020).

According to several studies, during the COVID-19 pandemic, events that generate stress, like fear of infection, having feelings of frustration, boredom, uncertainty, economic difficulties, psychological problems, or stigma and rejection toward infected people, physical and/or mental conditions, among others, have caused high levels of psychological, emotional, cognitive, and social imbalance to all age groups. Some human groups present a higher vulnerability when facing this extraordinary stressor. Such is the case with adolescent and young people, who require an additional effort to adapt. This population has suffered the highest impact of the restrictive measures due to lack socialization (Balluerka et al., 2020; Orte et al., 2020).

This lack of contact, and the particular situations triggered by the pandemic, increases the probability of psychological difficulties related to behavioral and emotional problems which manifest as stress, anxiety, or depression (Gómez-Becerra et al., 2020; Wang et al., 2020); therefore, big changes become vital stressors. Because of this, the COVID-19 pandemic and the lack of adequate psychological resources caused mental health problems and disorders (Sandín, 2003; Veytia et al., 2012; Villalobos et al., 2019).

During the months from March to June 2020 it was known that children and young people were showing a low infection risk from COVID-19. However, research shows that they are the most vulnerable to emotional discomfort (Orgilés et al., 2020). There is conclusive data showing that in this population there has been a great increase of psychiatric disorders such as anxiety, depression, and insomnia, especially in women who are close to infected people (Martínez-Taboas, 2020).

Nevertheless, this extraordinary vital situation has also brought prosocial behaviors. Carlo (2014) stated that these behaviors are related to voluntary behaviors like sharing, comforting, and helping. The mentioned behaviors occur in specific scenarios or situations and therefore different examples of prosocial behavior exist (Mestre et al., 2015). For some researchers, the situational and dispositional factors modulate prosocial behavior, explaining that, the higher the ambiguity and severity is in a specific situation, the higher the probability of the appearance of helping behaviors (Batson and Powell, 2003; Galen, 2012). This is the case, for example, of studies such as the one by van de Groep et al. (2020) who investigated the effect of the first weeks of the Covid-19 pandemic on mood, empathy, and prosocial behavior; the results suggest that need and deservedness had a greater influence on adolescent giving than familiarity in the ecologically valid context of the COVID-19 pandemic. Other studies had shown that after and during the early stage of the pandemic, individuals' general prosociality changed toward increased prosociality (Hellman et al., 2021; Yue and Yang, 2021).

Diverse research has shown that prosocial behavior has a highly significant relation to empathy. In fact, studies that empirically evaluate directly the psychological processes related to prosocial development highlight the important role of empathy as a motivator of prosocial behavior (Batson, 1998; Hoffman, 2000; Richaud and Mesurado, 2016), in its cognitive (perspective taking: ability to put oneself in the place of the other) and affective dimension (empathic concern: feelings oriented to the problem or need of another individual) (Knight et al., 2014; Van der Graaff et al., 2014). Research shows a consistent relationship between empathy and prosocial behavior as growth in empathy is associated with individual differences in prosocial behaviors in childhood (Eisenberg et al., 2014). According to Hoffman (1992), from the multidimensional perspective of empathy, prosociality has a psychological dimension to it, which generates an altruistic attitude in a person to help another in need. This dimension, combined with others such as the cognitive, affective, motivational, and spiritual dimension, contributes to respecting life, co-responsibility, solidarity, support, and resiliency in times of crisis (Boies, 2020). In this sense, studies have shown that empathy was positively associated with prosociality during the pandemic; this reveals that individuals with higher levels of empathy show more prosocial behaviors during the pandemic (Cho et al., 2021).

We can say then that an essential component in the development and appearance of thoughts and behaviors which are socially appropriate is empathy (Ventura, 2020). In fact, several authors maintain the idea that prosocial behaviors have an important function in social relations. Prosocial behavior and the related cognitive and emotional variables facilitate social interaction and adaptation. These behaviors have important consequences on health and social adjustment of individuals, especially adolescents and young people (Taylor et al., 2013; Llorca et al., 2017).

On the other hand, the way that a person assesses stressful events, as is the case of the pandemic, also has a direct impact over psychological adjustment. The assessment made of a stressful event can determine the consequences over mental health even more than the stressful event itself (Lazarus and Folkman, 1984). According to cognitive-relational theory, for an event to be stressful, first it must be perceived as such (Lazarus and Folkman, 1984). This theory is the process of evaluating the personal significance of events (Peacock and Wong, 1990). Primary appraisal involves an assessment of the importance of a transaction for one's wellbeing and include assessments of events and interactions as threats, challenges, and as central to oneself (Zacher and Rudolph, 2021). Threat appraisals involve the potential for harm/loss in the future and challenge appraisals reflect the anticipation of gain or growth from the experience. Challenge appraisals do not have the same negative implications that harm/loss or threat appraisals have, and can be positive or exciting for individuals (Oliver and Brough, 2002). Several studies have explored the different implications that experiencing stress as a challenge vs. as a threat has on other variables since the beginning of cognitive-relational theory such as, for example, that individuals with high levels of negative affectivity were more likely to appraise events as threatening, whereas that those with low levels of negative affectivity appraise them as a challenge (Gallager, 1990; Hemenover and Dienstbier, 1996) or most recently, that positive affect is positively related to challenge appraisals, and negatively related to threat appraisal and humor, and negative affect is positively related to threat appraisals, among other variables (Zacher and Rudolph, 2021). Furthermore, numerous studies show that there is a strong correlation between the assessment of a threat and the coping mechanisms which lead to a poor adaptation to the stressful situation. Whereas, the assessment of a challenge relates to a kind of efficient coping which allows the individual a greater state of wellbeing (Ramírez et al., 2008; Samper, 2014; Szkody and McKinney, 2020).

This pandemic situation has allowed us to consider values such as solidarity and cooperation and ethical and moral principles which specify that other people are important in our daily actions. In fact, the World Health Organization (WHO), to achieve its implicit objectives, affirms that mental health requires the gregarious character of humanity, that capacity to contribute to the community from the understanding that only together we will manage to reduce social inequalities and reach a good collective mental health. The voluntary acts of helping, sharing, and of commitment to others, actions that are distinctly human, become evident during crisis. These acts cause wellbeing (Villalobos, 2020).

Moreover, empirical studies conclude that prosociality and the ability to put oneself in the place of another are protective factors against impulsive responses and emotional instability when facing difficult situations that require from the subject a solution to a problem (Samper et al., 2015; Mestre et al., 2019). The capacity to discriminate and regulate emotions and to repair one's mood relates significantly with anxiety, anger, and depression (Salguero and Iruarrizaga, 2006). On the contrary, some authors have pointed out that the lack or the lessening of empathy has an influence on depression and anxiety (Caprara et al., 2010; Llorca et al., 2017). Emotional changes and mood changes are related to what is happening to us and to our experiences. That is why certain situations can lead to depression, anxiety, and other symptoms of distress (Moya, 2013; Llorca et al., 2014, 2017; Saladino et al., 2020). Therefore, these moments of crisis bring about the possibility to perform helping behaviors aimed to prevent and reduce the collective crisis. That is why with the present study we want to analyze the psychosocial effects of COVID-19, firstly, by presenting a cross-sectional study in an Ecuadorian population and secondly, a comparative study with two samples, namely an Ecuadorian population and Spanish population.

Hofstede (1980) considered Latin American countries to be more collectivist than European countries. Ecuador ranks amongst the most collectivistic cultures in the world, beaten only by Guatemala. Ecuadorians can show a lot of solidarity toward members of their in-groups. In comparison with other countries in Europe, Spain appears as collectivist. This has made Spaniards quite easy to relate with certain cultures -mainly non-European. However, compared with other areas of the world, Spanish culture clearly classes as individualistic (Mesurado et al., 2014). We have considered in our study these two countries because in April 2020, Ecuador had the highest levels of people infected by COVID-19 in South America, followed by Uruguay, Peru, Brazil, and Argentina, and was above the world average (Inca-Ruíz and Inca-León, 2020). That same month, Spain was, after the USA, the country with higher infection levels (Orte et al., 2020). With the declaration of the state of emergency due to the COVID-19 pandemic, commercial, educational, tourist, and land and air transport activities, among others, were closed. In Ecuador, intervention in health emergencies was inadequate and this was reflected in the mental health of Ecuadorians (Tusev et al., 2020). Existing research shows significant percentages of the prevalence of mental disorders in the population (adolescents and adults), with confinement due to COVID-19 being one of the causes of this situation (Velastegui et al., 2020; Catagua-Meza and Escobar-Delgado, 2021; Cifuentes-Carcelén and Navas-Cajamarca, 2021). Similarly in Spain, studies show that the confinement and the absence of schooling had a negative impact on the mental health of the child and adolescent population (Gatell-Carbó et al., 2021). Furthermore, the physical and mental health of the elderly was also being negatively affected, with an increase of sleep problems, sedentary lifestyle, and disorders due to anxiety and depression (Buitrago et al., 2021). With this comparative study we hope to be able to progress the understanding of the variable which helps to predict certain internalized problems across countries and cultures.

The objectives of the study are to describe how the Ecuadorian population felt (depression, anxiety, and stress) before and after confinement; analyze the effects that certain emotional and behavioral variables, such as prosocial tendencies, empathy, and experiencing stress as a challenge or as a threat, taken into consideration before confinement, have over depressive symptoms, anxiety, and stress perceived after confinement; and carry out a comparative study in a sample of the Ecuadorian and Spanish general populations.

Based on the literature and regarding the first objective, we hypothesize (hypothesis 1) that the levels of depression, anxiety, and stress will be higher after confinement (Orgilés et al., 2020; Xiang et al., 2020; Breaux et al., 2021; Rogers et al., 2021). Regarding the second objective, we hypothesize (hypothesis 2) that behaving in a prosocial manner, having a higher level of empathy, and experiencing stress as a challenge (not as a threat) will become a protector from depression symptoms, anxiety, and stress (Caprara et al., 2010; Llorca et al., 2014; Davis et al., 2016; Alarcón and Forbes, 2017). Finally, regarding the third objective, we hypothesize (hypothesis 3) that there will be no differences between the two countries in the effects of COVID-19 on depression symptoms, anxiety, and stress (Samji et al., 2021) but there will be differences regarding the effect that prosocial behavior, empathy, and assessing stress as a challenge or a threat will have on depressive symptoms, anxiety, and stress.

## Materials and Methods

### Participants

We present an analysis of the psychosocial effects of COVID-19 in two populations: first, a cross-sectional study in the Ecuadorian population (*n* = 301) and second, a comparative study between two samples from the Ecuadorian and Spanish populations (*n* = 83 each one). In the cross-sectional study, the Ecuadorian sample consisted of adolescents between 12 and 19 years of age (*M* = 16.14; *SD* = 1.93). As to gender, the sample consisted of 115 boys (38.2%) and 182 girls (60.5%). In relation to educational attainment, most of the students were studying baccalaureate and NVQ2 and Obligatory Secondary Education. They mostly indicate that they lived in a house in the center of the town, other lived in the outskirts of the city, or in the country or a village, in turn they perceived that they lived in a normal to very big space (71–120 m^2^) in a greater percentage, while the rest lived in a reduced space. Only 2.0% were living with a person who had had the virus, but 23.9% declared that a person close to them had had the virus (see Table 1 for details).

**Table 1:** Descriptive statistics.

	**Cross-sectional study**		**Comparative study**			
	**Ecuadorian sample**		**Ecuadorian sample**		**Spanish sample**	
	**(*****n*** **=** **301)**		**(*****n*** **=** **83)**		**(*****n*** **=** **83)**	
	***n***	**%**	***n***	**%**	***n***	**%**
**Gender**
Men	115	38.2	22	26.5	14	16.9
Women	182	60.5	59	71.1	67	80.7
Other	4	1.3	2	2.4		
**Educational attainment**
Baccalaureate and NVQ2	118	39.2	21	25.3	20	24.1
Obligatory secondary education	94	31.2	27	32.5	15	18.1
NVQ1	8	2.7	2	2.4	4	4.8
University degrees	64	21.3	29	34.9	41	49.3
**Geographic location**
In a house in the center of the town with a great number of neighbors	81	27.5	21	25.3	17	20.5
In a house in the center of the town with a few neighbors	46	15.3	12	14.5	24	28.9
In the outskirts of the city	93	30.9	28	33.8	14	16.8
In the country or a village	66	22.0	17	20.5	24	28.9
**Space**
In a normal to very big space (70–120 m^2^)	213	70.8	57	68.7	42	50.6
In a very big house	36	12.0	12	14.5	26	31.3
In a reduced or very reduced space	46	15.3	12	14.5	13	15.6
**Relation with people with COVID-19**
Participants who lived with a person with COVID-19	6	2.0	2	2.4	19	22.9
Participants who had close contact with a person with COVID-19	72	23.9	1	1.2	23	27.7

In the comparative study, the sample from Ecuador consisted of 83 subjects from 12 to 65 years of age (*M* = 34.41; *SD* = 15.17). Of this sample, 26.5% were men and 72.3% were women. The level of education comprises Baccalaureate and NVQs, secondary education, and university degrees. A majority of respondents indicated that they lived in a house in the center of the town, others lived in the outskirts of the city, while the rest lived in the country or a village. Most cases perceived that they lived in a normal to very big space (71–120 m^2^), while the rest lived in a reduced or very reduced space. Only 2.4% were living with a person who had had the virus, but 23.8% declared that a person close to them had had the virus. Furthermore, 83 subjects between 12 and 72 years old (*M* = 36.26; *SD* = 16.56) participated from Valencia, Spain, of which 16.9% were men and 80.7% were women. As with the Ecuadorian sample, the level of education comprises Baccalaureate and NVQs, secondary education, and university degrees. They mostly indicate that they lived in a house in the center of the town, and in the village. The rest live in the outskirts and in the country. However, the population evaluated mostly perceives that they live in a normal to very big space (71–120 m^2^), while the rest live in a reduced space. Moreover, only 1.2% of the sample evaluated lived with a person who had had COVID-19. However, 28.7% declared that a person close to them had had it (see Table 1 for details).

### Research Procedure

Participants completed an online survey through the Limey Survey platform which was available from May to June, 2020 (first wave of covid-19). First of all, the tests were selected based on the variables required and the psychometric properties. The procedure was changed to an online evaluation protocol through the LimeSuvey platform. Next, the pertinent licenses were obtained from the Ecuadorian Educational Coordination Zone 6 and the North District of the Cuenca canton, and the data collection process was carried out in some of the fiscal educational institutions of the City of Cuenca, motivating participants through the *Zoom* platform. At this time, it was indicated that the survey has three parts to be considered: in the first one they would find the signed consent; in the second one, they would find questions they should answer by thinking about how they were before the pandemic; and in the third, the same questions, but they should consider the actual extraordinary situation. Regarding the assessment of the Spanish sample, after obtaining the pertinent licenses, the battery of tests was sent through the LimeSurvey platform to public and private schools in the city of Valencia (Spain), as well as the public in general. We used different tactics to reach participants, relying on the social networks of the researchers, who reached out to social media audiences to broadcast and share the survey. The link was sent by email and two platforms (Facebook and WhatsApp) were used to disseminate the survey. A standardized general description about the survey was given in the email and messaging/social media postings. The participation was voluntary and anonymous, taking into consideration all ethical principles pertaining to research with human beings included in the Helsinki Declaration, under the current regulations.

### Measures

For the study of the selected variables, different batteries of questionnaires were used with an online format. Participants responded by thinking about the situation before and during/after confinement. The different items of the questionnaires were written using the appropriate verb tense to obtain answers in the two assessed times.

The assessment of the *sociodemographic questions* was carried out through an *ad hoc* questionnaire with questions related to gender, age, country of residence, level of studies, marital status, family socioeconomic situation, change in socioeconomic situation due to COVID-19, people in the household, place of residence, size of the dwelling, and finally, two questions to identify if any of the people in the household or close to the participant has had the virus.

*Prosocial tendencies* were evaluated with the Prosocial Tendencies Measure Revised (PTM-R) of Carlo et al. (2003) (Spanish adaptation by Mestre et al., 2015). This questionnaire evaluated different forms of prosocial behavior. It was composed of six subscales through 21 items, one for each of the following prosocial behaviors: public, emotional, altruism, anonymous, compliant, and dire. Participants responded to the items by choosing a response from a scale ranging from 1 (does not describe me at all) to 5 (describes me very well). The subject must describe their own behavior in a variety of situations that reflect different kinds of prosocial behavior: the subscale emotional (5 items) evaluated prosocial tendencies to help others in emotive situations (“It makes me feel good when I can comfort someone who is really distressed”); the subscale altruism (4 items) related to helping others when there is little or no chance of receiving an explicit, direct reward (“I believe that giving things or money is better if I obtain some benefit from it”); the subscale compliance (2 items) evaluated the tendency to help others when they ask for assistance (“I do not hesitate in helping people when they ask me to”); the subscale dire (3 items) measured prosocial behavior in dire situations or situations of crisis (“I have a tendency to help people in dire need”); the subscale public (3 items) measured behavior driven by an intention to behave prosocially in the presence of others (“I can help people better when others are looking at me”); finally, the subscale anonymous addressed the prosocial tendency to help strangers (“I have a tendency to help those in need when they do not know who is helping them”). Cronbach's alpha for all the main measures in the scale for this study, in both samples were: public: 0.75 Ecuador, 0.70 Spain; emotional: 0.81 Ecuador, 0.73 Spain; dire: 0.71 Ecuador, 0.70 Spain; anonymous: 0.81 Ecuador, 0.80 Spain; altruistic: 0.76 Ecuador, 0.74 Spain; and compliant: 0.71 Ecuador, 0.70 Spain.

To evaluate *Depression, Anxiety*, and *Stress*, DASS-21 (Spanish adaptation by Daza et al., 2002; Norton, 2007) has been used in its abbreviated version (originally 42 items). Each of the three scales contain seven items. The subscale *depression*, characterized by the loss of self-esteem and the incentive to reach vital goals, is evaluated through items like: “I couldn't/I haven't been able to feel any positive feelings” (depression; α = 0.83); *anxiety* was evaluated through descriptions related to physical symptoms of excitement, panic attack, muscle tension, and fear through statements like: “I noticed/I have noticed that my mouth was dry” (anxiety; α = 0.79); stress, the tendency to react with tension, irritability, and persistent activation when facing stressful situations was evaluated through descriptions like: “I found/I have found it very difficult to relax” (stress; α = 0.84). The answers were evaluated with a 4-point scale from 0 (It did not occur to me) to 3 (It occurred most of the time). The participants were asked to answer with what frequency they experienced these sensations before the pandemic and during/after the pandemic. Cronbach's alpha for all the main measures in the scale for this study, in both samples were: depression: 0.84 Ecuador, 0.83 Spain; anxiety: 0.90 Ecuador, 0.86 Spain; and stress: 0.90 Ecuador, 0.88 Spain.

The Stress Appraisal Measure (SAM-A) was used to assess *stress* (Rowley et al., 2005; Spanish adaptation by our research team). Rowley et al. (2005) affirm that in their day-to-day life, people show certain dispositional tendencies to evaluate stressful factors and therefore respond to them in a particular way. The instrument consists of two subscales that assess *stress as a challenge* (4 items), which refers to the person's ability to assess either the harm or the potential benefit that may result from a particular situation and the person tends to overcome it and achieve their goals, with items like: “I considered I have/have had the ability to overcome stress;” and *stress as a threat* (7 items), which is related to the tendency to normally evaluate stressful events as threatening, which paralyzes the positive action of the subject. This tendency is measured with items like: “I perceived/Have perceived stress as a threat.” Moreover, this instrument includes three items that represent a *secondary assessment*, namely, the ability to assess what can be done in order to face or benefit from a situation which is causing distress so that the subject is able to bring to bear their personal resources to reduce the potential harm or improve the possibility of benefit (Folkman et al., 1986). This is measured with items like: “I considered that/There are people I can/I have been able to ask for help.” The answers were assessed with a 5-point scale: 0 = nothing; 1 = a little; 2 = some; 3 = enough; 4 = a lot (Rowley et al., 2005). Cronbach's alpha for all the main measures in the scale for this study in both samples were: stress as a challenge: 0.86 Ecuador, 0.85 Spain; stress as a threat: 0.88 Ecuador, 0.90 Spain.

The Multidimensional Evaluation of Sympathy for adolescents by Richaud et al. (2017) was used to evaluate *empathy* from a social-cognitive perspective which represents three components which show: (1) the affective response to others' emotions and actions, (2) the cognitive process to the affective response, and (3) the conscious decision making to undertake an empathetic or prosocial action (Decety and Jackson, 2004; Decety and Lamm, 2006). According to the model subjacent in the instrument, the coincidence of the distress the other person is experiencing leads to solidarity and/or altruism (Lietz et al., 2011). Each factor is assessed through three items. The *self-awareness* factor was measured through items such as: “I noticed/I have noticed quickly when someone *felt*/ has felt badly” (α = 0.75). This refers to the ability to identify what the subject felt at the moment of affective excitement and at the same time evoke thoughts and feelings of others (Lamm et al., 2007). *Perspective taking* was evaluated through items like: “Even if another person thinks differently to me, I *could*/have been able to understand them” (α = 0.72). This implies noticing that another person exists. *Emotional regulation*, a complex cognitive process, is related to the ability to change one's way of thinking which influences the way of feeling, and is analyzed through items like: “I *had*/have had outbursts of anger” (α = 0.72). *Emotional contagion*, the dimension that allows one to emotionally respond due to the recognition and understanding of the emotional state of another person, is evaluated with questions like: “*When I saw someone crying*/When I have seen someone, I don't know crying, I have felt like crying” (α = 0.78). *Empathetic action* as the ability to carry out empathetic behaviors is assessed through: “*I thought* / I have thought that everyone should help those in need” (α = 0.70). The answers are assessed with a 5-point scale (1 = never; 2 = a few times; 3 = many times; and 4 = always). Cronbach's alpha for all the main measures in the scale for this study, in both samples, were: *self-awareness*: 0.72 Ecuador, 0.70 Spain; *Perspective taking*: 0.62 Ecuador, 0.76 Spain; *Emotional regulation*: 79 Ecuador, 0.83 Spain; *Emotional contagion*: 0.76 Ecuador, 0.68 Spain; *Empathetic action*: 0.75 Ecuador, 0.81 Spain.

### Statistical Procedure

Firstly, SPSS 26 was used to estimate means and standard deviations and to calculate repeated measures analysis of variance (ANOVA) to test for mean differences across waves (before and during/after confinement) and countries, Ecuador and Spain. Secondly, multiple linear regressions in steps were carried out with the Ecuadorian population and according to the country, Ecuador and Spain, to analyze the predictive value of the different psychological variables studied. The dependent variables are depression, anxiety, and stress during/after confinement, and the independent variables are prosocial behaviors, depression, anxiety, stress, stress challenge, stress threat, and reactive and proactive aggression. Collinearity analysis reveals that the data is free from problems of this nature. The condition index stands at values of <30 and the proportion of decomposition of variance in proportions of <0.5 (Belsley, 1991).

## Results

Firstly, to answer the first objective, a repeated measures analysis was carried out with the finality to study the differences among the variables assessed in the situation before and during/after confinement. The variables analyzed were depression, anxiety, and stress in Ecuadorian adolescents. Table 2 presents means, standard deviations, and results for the repeated measures analysis of variance (ANOVA) testing mean differences across the two time points (before and after confinement).

**Table 2:** Repeated measure analysis of depression, anxiety, and stress according to the situation before and during/after confinement.

	**Before confinement**		**After confinement**				
	***M***	***DT***	***M***	***DT***	***F***	***p***	***d***
Depression	0.97	0.88	1.10	0.78	13.79	0.000	0.16
Anxiety	0.82	0.80	0.89	0.78	4.48	0.035	0.08
Stress	1.04	0.83	1.26	0.81	37.19	0.000	0.27
Public	2.40	1.13	2.86	1.04	74.20	0.000	0.42
Emotional	3.63	0.98	3.87	0.83	25.46	0.000	0.26
Altruistic	3.77	1.08	3.49	1.10	31.60	0.000	0.25
Dire	3.49	1.02	3.43	0.94	0.91	0.338	0.06
Compliant	3.81	1.06	3.87	1.03	0.76	0.380	0.05
Anonymous	3.23	1.11	3.32	1.02	3.48	0.063	0.08
Stress challenge	2.15	1.16	2.20	1.03	0.67	0.412	0.04
Stress threat	1.78	1.11	1.84	1.09	1.02	0.311	0.05
Secondary assessment	2.35	1.21	1.99	1.22	28.46	0.000	0.29
Emotional contagion	2.51	0.78	2.56	0.72	1.05	0.312	0.04
Empathetic action	3.04	0.78	3.25	0.62	30.39	0.000	0.62
Perspective taking	2.82	0.79	2.93	0.69	8.28	0.004	0.14
Emotional regulation	2.13	0.87	2.43	0.88	48.97	0.000	0.34
Self-awareness	3.00	0.83	3.06	0.72	2.15	0.143	0.07

The differences were significant for all dependent variables: depression (*F* = 13.79, *p* = 0.000), anxiety (*F* = 4.48, *p* = 0.035), and stress (*F* = 37.19, *p* = 0.000). The scores increased significantly after the pandemic. The effect size in stress is medium (Cohen's *d* = 0.27), whereas in depression and anxiety, the effect size of both variables is small (Cohen's *d* = 0.16, and 0.08, respectively) (Cohen, 1988). In relation to the independent variables, the differences were significant for public (*F* = 74.20, *p* = 0.000), emotional (*F* = 25.46, *p* = 0.000), and altruistic (*F* = 31.60, *p* = 0.000) prosocial behaviors, secondary assessment (*F* = 28.46, *p* = 0.000), empathetic action (*F* = 30.39, *p* = 0.000), perspective taking (*F* = 8.28, *p* = 0.000), and emotional regulation (*F* = 48.97, *p* = 0.000). The scores raised significantly after the pandemic for public and emotional prosocial behaviors and for all variables related to empathy. Furthermore, the scores diminished significantly after the pandemic for altruistic prosocial behavior and secondary assessment. The effect size in empathic action is large (Cohen's *d* = 0.62), in public, emotional, and altruistic prosocial behaviors, secondary assessment and emotional regulation is medium (Cohen's *d* = 0.42, 0.26, 0.25, 0.29, and 0.34, respectively), whereas in perspective taking (Cohen's *d* = 0.14), the effect size is small (Cohen, 1988). Bivariate correlations for all study variables are shown in Tables 3, 4; see *Supplementary materials* for details.

**Table 3:** Correlation matrix for the study variables in the ecuadorian sample (*n* = 301).

**Variable**	**1**	**2**	**3**	**4**	**5**	**6**	**7**	**8**	**9**	**10**	**11**	**12**	**13**	**14**	**15**	**16**	**17**	**18**	**19**	**20**	**21**	**22**	**23**	**24**	**25**	**26**	**27**	**28**	**29**	**30**	**31**
1. Depression (A)	–																														
2. Anxiety (A)	0.82^**^	–																													
3. Stress (A)	0.82^**^	0.86^**^	–																												
4. Public (B)	0.17^**^	0.24^**^	0.19^**^	–																											
5. Emotional (B)	0.13^*^	0.16^**^	0.18^**^	0.30^**^	–																										
6. Altruistic (B)	−0.16^**^	−0.16^**^	−0.0.11^*^	−0.75^**^	−0.15^**^	–																									
7. Dire (B)	0.08	0.14^*^	0.13^*^	0.31^**^	0.72^**^	−0.18^**^	–																								
8. Compliant (B)	0.09	0.11	0.16^**^	0.10	0.67^**^	0.02	0.66^**^	–																							
9. Anonymous (B)	0.04	0.06	0.09	0.20^**^	0.52^**^	−0.16^**^	0.60^**^	0.50^**^	–																						
10. Public (A)	0.10	0.13^*^	0.10	0.63^**^	0.25^**^	−0.53^**^	0.23^**^	0.00	0.08	–																					
11. Emotional (A)	0.14^*^	0.16^**^	0.18^**^	0.21^**^	0.58^**^	−0.14^*^	0.46^**^	0.35^**^	0.25^**^	0.35^**^	–																				
12. Altruistic (A)	−0.16^**^	−0.09	−0.05	−0.61^**^	−0.03	0.68^**^	−0.06	0.11^*^	−0.00	−0.58^**^	−0.13^*^	–																			
13. Dire (A)	0.05	0.13^*^	0.09	0.22^**^	0.37^**^	−0.19^**^	0.49^**^	0.35^**^	0.37^**^	0.37^**^	0.50^**^	−0.18^**^	–																		
14. Compliant (A)	−0.01	0.05	0.09	0.09	0.35^**^	−0.02	0.35^**^	0.45^**^	0.19^**^	0.18^**^	0.52^**^	0.07	0.35^**^	–																	
15. Anonymous (A)	0.04	0.09	0.10	0.09	0.29^**^	−0.07	0.37^**^	0.31^**^	0.65^**^	0.08	0.39^**^	−0.01	0.49^**^	0.36^**^	–																
16. Stress challenge (B)	−0.11	−0.03	0.01	0.02	0.25^**^	0.03	0.23^**^	0.27^**^	0.24^**^	−0.03	0.03	0.15^**^	0.04	0.16^**^	0.11	–															
17. Stress threat (B)	0.48^**^	0.50^**^	0.56^**^	0.19^**^	0.16^**^	−0.05	0.12^*^	0.18^**^	0.16^**^	0.01	0.07	0.01	0.09	0.09	0.12^*^	0.27^**^	–														
18. Secondary assessment (B)	−0.12^*^	−0.05	−0.00	0.04	0.24^**^	0.02	0.23^**^	0.23^**^	0.22^**^	0.03	0.05	0.08	0.13^*^	0.10	0.11	0.68^**^	0.25^**^	–													
19. Stress challenge (A)	−0.16^**^	−0.06	−0.05	−0.00	0.18^**^	−0.04	0.26^**^	0.24^**^	0.21^**^	0.06	0.07	0.06	0.12^*^	0.20^**^	0.15^**^	0.51^**^	0.02	0.43^**^	–												
20. Stress threat (A)	0.64^**^	0.64^**^	0.72^**^	0.15^**^	0.20^**^	−0.07	0.12^*^	0.19^**^	0.15^**^	−0.02	0.17^**^	0.01	0.09	0.09	0.13^*^	0.09	0.68^**^	0.10	−0.00	–											
21. Secondary assessment (A)	−0.11	0.01	0.02	0.04	0.22^**^	−0.02	0.29^**^	0.23^**^	0.23^**^	0.09	0.19^**^	0.04	0.24^**^	0.18^**^	0.19^**^	0.35^**^	0.10	0.53^**^	0.48^**^	0.11^*^	–										
22. Emotional contagion (B)	0.29^**^	0.34^**^	0.35^**^	0.15^**^	0.36^**^	−0.03	0.28^**^	0.29^**^	0.22^**^	0.15^**^	0.27^**^	−0.04	0.17^**^	0.16^**^	0.15^**^	0.09	0.32^**^	0.15^**^	0.13^*^	0.34^**^	0.23^**^	–									
23. Empathetic action (B)	0.06	0.081	0.13^*^	0.08	0.55^**^	0.09	0.49^**^	0.53^**^	0.38^**^	0.02	0.32^**^	0.11^*^	0.20^**^	0.29^**^	0.19^**^	0.41^**^	0.20^**^	0.36^**^	0.22^**^	0.16^**^	0.15^**^	0.28^**^	–								
24. Perspective taking (B)	0.12^*^	0.13^*^	0.19^**^	0.06	0.42^**^	0.04	0.38^**^	0.43^**^	0.30^**^	0.02	0.19^**^	0.13^*^	0.17^**^	0.24^**^	0.13^*^	0.46^**^	0.26^**^	0.41^**^	0.30^**^	0.17^**^	0.25^**^	0.36^**^	0.60^**^	–							
25. Emotional regulation (B)	0.55^**^	0.54^**^	0.58^**^	0.27^**^	0.20^**^	−0.23^**^	0.16^**^	0.05	0.15^**^	0.19^**^	0.17^**^	−0.16^**^	0.16^**^	0.02	0.15^**^	−0.04	0.53^**^	−0.01	−0.06	0.48^**^	−0.05	0.31^**^	0.19^**^	0.16^**^	–						
26. Self-Awareness (B)	0.14^**^	0.12^*^	0.17^**^	0.04	0.48^**^	0.08	0.39^**^	0.46^**^	0.33^**^	−0.01	0.24^**^	0.17^**^	0.14^*^	0.24^**^	0.17^**^	0.40^**^	0.28^**^	0.39^**^	0.23^**^	0.22^**^	0.15^**^	0.25^**^	0.67^**^	0.64^**^	0.20^**^	–					
27. Emotional contagion (A)	0.29^**^	0.34^**^	0.35^**^	0.15^**^	0.36^**^	−0.03	0.28^**^	0.29^**^	0.22^**^	0.15^**^	0.27^**^	−0.04	0.17^**^	0.16^**^	0.15^**^	0.09	0.32^**^	0.15^**^	0.13^*^	0.34^**^	0.23^**^	0.66^**^	0.28^**^	0.36^**^	0.31^**^	0.25^**^	–				
28. Empathetic action (A)	0.06	0.13^*^	0.15^**^	0.14^*^	0.54^**^	0.05	0.51^**^	0.54^**^	0.34^**^	0.14^*^	0.43^**^	0.06	0.31^**^	0.39^**^	0.27^**^	0.16^**^	0.16^**^	0.24^**^	0.21^**^	0.12^*^	0.23^**^	0.40^**^	0.58^**^	0.36^**^	0.10	0.37^**^	0.40^**^	–			
29. Perspective taking (A)	0.08	0.17^**^	0.19^**^	0.10	0.37^**^	−0.06	0.37^**^	0.37^**^	0.22^**^	0.15^**^	0.26^**^	0.04	0.24^**^	0.32^**^	0.15^**^	0.30^**^	0.18^**^	0.30^**^	0.37^**^	0.16^**^	0.31^**^	0.24^**^	0.35^**^	0.62^**^	0.07	0.40^**^	0.24^**^	0.44^**^	–		
30. Emotional regulation (A)	0.59^**^	0.56^**^	0.63^**^	0.23^**^	0.19^**^	−0.19^**^	0.14^*^	0.12^*^	0.11^*^	0.12^*^	0.19^**^	−0.15^**^	0.10	0.00	0.12^*^	−0.12^*^	0.43^**^	−0.08	−0.11^*^	0.56^**^	−0.08	0.37^**^	0.11^*^	0.08	0.62^**^	0.11^*^	0.37^**^	0.18^**^	0.04	–	
31. Self-Awareness (A)	0.25^**^	0.22^**^	0.27^**^	0.03	0.40^**^	0.06	0.37^**^	0.36^**^	0.27^**^	0.07	0.28^**^	0.09	0.20^**^	0.21^**^	0.20^**^	0.27^**^	0.29^**^	0.31^**^	0.20^**^	0.29^**^	0.27^**^	0.37^**^	0.43^**^	0.53^**^	0.18^**^	0.64^**^	0.37^**^	0.49^**^	0.51^**^	0.28^**^	–

*B, before confinement; A, after confinement*.

* *p < 0.05*;

** *p < 0.01*.

**Table 4:** Correlation matrix for the study variables by country, Ecuador (*n* = 83) and Spain (*n* = 83).

**Variable**	**1**	**2**	**3**	**4**	**5**	**6**	**7**	**8**	**9**	**10**	**11**	**12**	**13**	**14**	**15**	**16**	**17**	**18**	**19**	**20**	**21**	**22**	**23**	**24**	**25**	**26**	**27**	**28**	**29**	**30**	**31**
1. Depression (A)	–	0.75^**^	0.72^**^	**0.13**	**0.01**	−0.30^**^	**0.03**	**−0.13**	**0.08**	**0.09**	**0.10**	−0.36^**^	**0.17**	**−0.05**	**0.03**	−0.27*	0.32^**^	**−0.18**	−0.28^**^	0.61^**^	**−0.05**	0.29^**^	**−0.08**	**−0.02**	0.35^**^	**0.00**	0.29^**^	**0.04**	**0.03**	0.61^**^	**0.04**
2. Anxiety (A)	0.87^**^	–	0.78^**^	**0.16**	**0.13**	−0.22*	**0.12**	**−0.05**	**0.10**	**0.05**	**0.08**	−0.22*	0.22*	**−0.04**	**0.03**	**−0.19**	0.31^**^	**−0.03**	**−0.09**	0.61^**^	**0.02**	0.31^**^	**0.08**	**0.06**	0.33^**^	**0.13**	0.31^**^	**0.04**	**0.04**	0.59^**^	**0.08**
3. Stress (A)	0.84^**^	0.87^**^	–	**−0.04**	**0.18**	**−0.15**	**0.06**	**0.04**	**0.20**	**−0.13**	**0.03**	**−0.05**	**0.03**	**0.01**	**0.07**	**−0.14**	0.45^**^	**−0.07**	**−0.11**	0.67^**^	**−0.08**	0.28*	**−0.03**	**0.04**	0.42^**^	**0.06**	0.28*	**−0.01**	**0.00**	0.65^**^	**0.07**
4. Public (B)	0.32^**^	0.32^**^	0.31^**^	–	**−0.02**	−0.70^**^	**0.15**	**−0.12**	**−0.06**	0.28^**^	**0.03**	−0.55^**^	**0.12**	**−0.12**	**0.03**	**−0.04**	**−0.09**	**−0.01**	**−0.09**	**−0.01**	**0.03**	**−0.16**	**−0.10**	**−0.11**	0.25*	**0.02**	**−0.16**	**−0.10**	**−0.08**	**0.06**	**−0.14**
5. Emotional (B)	0.24^*^	0.21	0.21	0.33^**^	–	**0.04**	0.67^**^	0.75^**^	0.39^**^	**−0.11**	0.72^**^	**−0.11**	0.29^**^	0.42^**^	0.45^**^	0.44^**^	0.28*	0.48^**^	0.40^**^	**0.20**	0.32^**^	0.45^**^	0.59^**^	0.23*	**0.17**	0.45^**^	0.45^**^	0.49^**^	0.31^**^	**0.21**	0.42^**^
6. Altruistic (B)	−0.21	−0.23^*^	−0.21	−0.81^**^	−0.24^*^	–	**−0.12**	0.25*	**−0.09**	−0.33^**^	**−0.03**	0.59^**^	**−0.08**	**0.14**	**−0.08**	0.29^**^	**0.01**	0.25*	0.28^**^	**−0.13**	**0.21**	**0.11**	0.22*	**0.21**	−0.39^**^	**0.09**	**0.11**	**0.234**	0.24*	−0.25*	**0.20**
7. Dire (B)	0.15	0.20	0.18	0.32^**^	0.79^**^	−0.22^*^	–	0.58^**^	0.41^**^	**0.06**	0.46^**^	−0.26*	0.30^**^	0.28*	0.47^**^	0.35^**^	**0.17**	0.50^**^	0.24*	**0.20**	0.29^**^	**0.18**	0.49^**^	0.22*	0.23*	0.39^**^	**0.18**	0.32^**^	**0.17**	**0.09**	0.34^**^
8. Compliant (B)	0.17	0.13	0.16	0.14	0.61^**^	−0.09	0.68^**^	–	0.22*	**−0.15**	0.49^**^	**0.03**	**0.11**	0.51^**^	**0.21**	0.62^**^	**0.18**	0.54^**^	0.48^**^	**0.09**	0.31^**^	0.43^**^	0.66^**^	0.39^**^	**0.08**	0.52^**^	0.43^**^	0.60^**^	0.41^**^	**0.07**	0.63^**^
9. Anonymous (B)	0.13	0.05	0.10	0.15	0.51^**^	−0.11	0.60^**^	0.54^**^	–	**0.07**	0.30^**^	**−0.14**	**0.19**	**−0.02**	0.63^**^	**0.07**	0.47^**^	**0.11**	**0.11**	**0.20**	**0.03**	**−0.08**	**0.18**	**0.16**	**0.18**	**0.14**	**−0.08**	**0.15**	**0.08**	0.24*	**0.17**
10. Public (A)	0.17	0.21	0.21	0.66^**^	0.21	−0.59^**^	0.22^*^	−0.04	0.01	–	**0.10**	−0.51^**^	**0.17**	**−0.05**	**0.08**	**−0.08**	**−0.12**	**−0.12**	−0.30^**^	**−0.10**	**−0.09**	**−0.13**	**−0.13**	**−0.12**	**0.06**	**−0.08**	**−0.13**	**−0.12**	**−0.05**	**−0.03**	**0.02**
11. Emotional (A)	0.24^*^	0.28^**^	0.22^*^	0.30^**^	0.62^**^	−0.26^*^	0.55^**^	0.24^*^	0.29^**^	0.40^**^	–	−0.22*	0.49^**^	0.41^**^	0.37^**^	0.33^**^	**0.12**	0.31^**^	0.25*	**0.09**	0.34^**^	0.29^**^	0.40^**^	**0.15**	**0.10**	0.36^**^	0.29^**^	0.41^**^	0.39^**^	**0.19**	0.33^**^
12. Altruistic (A)	−0.27^*^	−0.21	−0.21^*^	−0.71^**^	−0.13	0.68^**^	−0.12	0.03	0.01	−0.68^**^	−0.26^*^	–	**−0.12**	**0.19**	**−0.20**	**0.03**	**−0.05**	**0.01**	0.22*	**−0.05**	**−0.02**	**0.10**	**0.04**	**0.10**	−0.25*	**−0.11**	**0.10**	**0.06**	**0.00**	**−0.17**	**−0.10**
13. Dire (A)	0.13	0.22^*^	0.21	0.23^*^	0.45^**^	−0.22^*^	0.59^**^	0.27^*^	0.37^**^	0.42^**^	0.68^**^	−0.19	–	0.23*	0.42^**^	**0.08**	**0.08**	**0.16**	**0.09**	**0.21**	**0.16**	**0.15**	0.28^**^	**0.18**	**0.15**	0.29^**^	**0.15**	0.30^**^	**0.21**	**0.10**	**0.15**
14. Compliant (A)	0.08	0.21	0.17	0.02	0.21	−0.07	0.39^**^	0.35^**^	0.17	0.20	0.49^**^	−0.02	0.56^**^	–	**−0.08**	0.44^**^	**−0.01**	0.36^**^	0.23*	**0.04**	**0.07**	0.26*	0.37^**^	**0.21**	**0.08**	0.27*	0.26*	0.43^**^	0.33^**^	**0.11**	0.35^**^
15. Anonymous (A)	0.07	0.10	0.07	0.13	0.29^**^	−0.09	0.44^**^	0.28^**^	0.64^**^	0.08	0.50^**^	−0.03	0.57^**^	0.46^**^	–	**0.16**	0.44^**^	**0.19**	0.25*	**0.13**	**0.10**	**−0.04**	0.35^**^	0.29^**^	**0.19**	0.27*	**−0.04**	0.34^**^	**0.12**	**−0.02**	**0.21**
16. Stress challenge (B)	−0.11	0.03	0.00	0.04	0.18	−0.06	0.31^**^	0.33^**^	0.23^*^	0.04	0.02	0.02	0.13	0.13	0.08	–	**0.06**	0.83^**^	0.63^**^	**−0.08**	0.45^**^	**0.16**	0.57^**^	0.54^**^	**−0.16**	0.63^**^	**0.16**	0.41^**^	0.45^**^	**−0.19**	0.60^**^
17. Stress threat (B)	0.52^**^	0.55^**^	0.54^**^	0.34^**^	0.28^**^	−0.26^*^	0.28^**^	0.33^**^	0.21	0.12	0.25^*^	−0.18	0.23^*^	0.16	0.21^*^	0.21	–	**0.11**	**0.17**	0.57^**^	**0.10**	**0.20**	**0.20**	0.29^**^	0.33^**^	0.21*	**0.20**	0.22*	0.30^**^	0.47^**^	0.25*
18. Secondary assessment (B)	−0.11	0.03	0.08	0.17	0.31^**^	−0.13	0.42^**^	0.41^**^	0.31^**^	0.05	0.09	0.02	0.23^*^	0.10	0.16	0.77^**^	0.24^*^	–	0.59^**^	**−0.02**	0.65^**^	0.23*	0.57^**^	0.52^**^	**−0.17**	0.58^**^	0.23*	0.37^**^	0.42^**^	**−0.17**	0.51^**^
19. Stress challenge (A)	−0.23^*^	−0.08	−0.16	0.01	0.13	−0.08	0.29^**^	0.25^*^	0.18	0.14	−0.02	0.06	0.21	0.24^*^	0.16	0.54^**^	0.01	0.51^**^	–	**0.03**	0.37^**^	0.24*	0.42^**^	0.45^**^	**−0.13**	0.39^**^	0.24*	0.45^**^	0.34^**^	**−0.18**	0.37^**^
20. Stress threat (A)	0.69^**^	0.68^**^	0.69^**^	0.19	0.17	−0.18	0.13	0.22^*^	0.12	−0.04	0.21	−0.09	0.12	0.17	0.21^*^	0.01	0.66^**^	0.06	−0.15	–	**0.07**	0.46^**^	**0.04**	**0.07**	0.33^**^	**0.18**	0.46^**^	**0.17**	**0.21**	0.65^**^	**0.21**
21. Secondary assessment (A)	−0.03	0.19	0.15	0.05	0.22^*^	−0.04	0.39^**^	0.20	0.27^*^	0.13	0.26^*^	0.13	0.37^**^	0.27^*^	0.32^**^	0.41^**^	0.21	0.57^**^	0.44^**^	0.22^*^	–	0.29^**^	0.34^**^	0.28^**^	**−0.21**	0.40^**^	0.29^**^	0.31^**^	0.46^**^	**−0.07**	0.36^**^
22. Emotional contagion (B)	0.40^**^	0.49^**^	0.41^**^	0.30^**^	0.35^**^	−0.22^*^	0.33^**^	0.34^**^	0.25^*^	0.20	0.21^*^	−0.24^*^	0.07	0.06	0.17	0.15	0.40^**^	0.14	0.08	0.37^**^	0.20	–	0.41^**^	0.23*	**0.15**	0.35^**^	0.70^**^	0.47^**^	0.41^**^	0.40^**^	0.39^**^
23. Empathetic action (B)	0.16	0.14	0.21	0.10	0.53^**^	0.07	0.54^**^	0.70^**^	0.44^**^	−0.06	0.18	0.06	0.17	0.21^*^	0.19	0.36^**^	0.28^**^	0.42^**^	0.21	0.19	0.19	0.25^*^	–	0.60^**^	**0.02**	0.71^**^	0.41^**^	0.68^**^	0.43^**^	**0.05**	0.58^**^
24. Perspective taking (B)	0.04	0.13	0.17	0.15	0.42^**^	−0.02	0.49^**^	0.58^**^	0.35^**^	0.05	0.16	0.09	0.21	0.21^*^	0.13	0.52^**^	0.24^*^	0.59^**^	0.31^**^	0.12	0.36^**^	0.34^**^	0.69^**^	–	**0.00**	0.60^**^	0.23*	0.40^**^	0.65^**^	**0.01**	0.49^**^
25. Emotional regulation (B)	0.57^**^	0.53^**^	0.56^**^	0.33^**^	0.23^*^	−0.34^**^	0.12	0.09	0.17	0.21	0.20	−0.25^*^	0.20	−0.03	0.12	−0.11	0.56^**^	−0.02	−0.21^*^	0.42^**^	0.03	0.27^*^	0.16	0.08	–	**0.13**	**0.15**	**0.08**	**0.07**	0.55^**^	**0.05**
26. Self-Awareness (B)	0.11	0.10	0.21	0.10	0.51^**^	0.03	0.54^**^	0.58^**^	0.48^**^	−0.09	0.24^*^	0.19	0.24^*^	0.22^*^	0.31^**^	0.24^*^	0.29^**^	0.45^**^	0.20	0.22^*^	0.24^*^	0.23^*^	0.71^**^	0.62^**^	0.27^*^	–	0.35^**^	0.52^**^	0.48^**^	**0.15**	0.74^**^
27. Emotional contagion (A)	0.40^**^	0.49^**^	0.41^**^	0.30^**^	0.35^**^	−0.22^*^	0.33^**^	0.34^**^	0.25^*^	0.20	0.21^*^	−0.24^*^	0.07	0.06	0.17	0.15	0.40^**^	0.14	0.08	0.37^**^	0.20	0.66^**^	0.25^*^	0.34^**^	0.27^*^	0.23^*^	–	0.47^**^	0.41^**^	0.40^**^	0.39^**^
28. Empathetic action (A)	0.23^*^	0.21	0.25^*^	0.20	0.60^**^	−0.08	0.63^**^	0.69^**^	0.54^**^	0.13	0.33^**^	−0.07	0.28^**^	0.31^**^	0.35^**^	0.18	0.30^**^	0.29^**^	0.25^*^	0.21	0.29^**^	0.41^**^	0.71^**^	0.54^**^	0.18	0.53^**^	0.41^**^	–	0.54^**^	**0.12**	0.68^**^
29. Perspective taking (A)	−0.01	0.16	0.13	0.04	0.33^**^	−0.04	0.40^**^	0.35^**^	0.16	0.15	0.08	0.15	0.25^*^	0.22^*^	0.09	0.31^**^	0.13	0.41^**^	0.49^**^	0.07	0.45^**^	0.34^**^	0.44^**^	0.69^**^	0.01	0.42^**^	0.34^**^	0.48^**^	–	0.24*	0.60^**^
30. Emotional regulation (A)	0.61^**^	0.53^**^	0.60^**^	0.32^**^	0.244	−0.33^**^	0.13	0.17	0.09	0.18	0.24^*^	−0.32^**^	0.10	0.06	0.10	−0.12	0.45^**^	−0.06	−0.24^*^	0.53^**^	−0.03	0.36^**^	0.17	−0.02	0.60^**^	0.17	0.36^**^	0.22^*^	−0.09	–	**0.15**
31. Self-Awareness (A)	0.24^*^	0.25^*^	0.33^**^	0.04	0.50^**^	0.01	0.51^**^	0.45^**^	0.40^**^	0.15	0.24^*^	0.10	0.29^**^	0.25^*^	0.26^*^	0.24^*^	0.26^*^	0.37^**^	0.24^*^	0.28^*^	0.48^**^	0.42^**^	0.44^**^	0.51^**^	0.27^*^	0.61^**^	0.42^**^	0.56^**^	0.58^**^	0.24^*^	–

*B, before confinement; A, after confinement*.

* *p < 0.05*;

** *p < 0.01. Spanish sample = in bold*.

Secondly, to answer the second objective, we computed three multiple linear regressions to gain insight into the predictive variables of depression, anxiety, and stress during/after confinement, from Ecuadorian adolescents. In addition, we analyzed how all variables were inter-correlated with each other by computing Pearson correlations (see Tables 3, 4).

The pattern of correlations observed in all samples indicates that, in general, depression, anxiety, and stress experienced by participants of all three samples after confinement correlate directly and significantly with stress threat and with emotional contagion and emotional regulation (experienced before and after confinement). Conversely, the correlation is inverse and significant in all three samples with altruistic prosocial behavior. Furthermore, with the two Ecuadorians samples, the correlation is positive and significant with public and emotional prosocial behavior and with self-awareness, while depression correlates inversely and significantly with stress challenge.

Regression analysis for Ecuadorian adolescents (Table 5) showed that for depression, 53.7% of the variance was explained by public prosocial behavior (*B* = 0.003), stress challenge (*B* = −0.08), and threat (*B* = 0.020) and emotional regulation (before confinement) (*B* = 0.18), and stress threat (*B* = 0.30) and emotional regulation (after confinement) (*B* = 0.17). For anxiety, 52.2% (*R*^2^ = 0.52) was explained by the variables relating to public prosocial behavior (*B* = 0.04), stress challenge (*B* = −0.05) and threat (*B* = 0.02), and emotional regulation (before confinement) (*B* = 0.17), and stress threat (*B* = 0.30), emotional regulation (*B* = 0.14), and perspective taking (*B* = 0.10) (after confinement). Finally, for stress, 63% (*R*^2^ = 0.63) is explained by the variables relating to public prosocial behavior (*B* = 0.003) and emotional regulation (*B* = 0.18) (before confinement), and emotional prosocial behavior (*B* = −0.001), stress threat (*B* = 0.36), emotional regulation (*B* = 0.21), and perspective taking (*B* = 0.10) (after confinement). The variables stress challenge and emotional prosocial behavior have negative relations with the dependent variables, while all other variables have positive relations.

**Table 5:** Multiple linear regression analysis in Ecuador.

	***R* squared**	***B***	**Standard error**	**Beta**	***t***	**Sig**.
**Depression after confinement**
Constant		−0.146	0.127		−1.155	
Public (B)	0.031	0.003	0.029	0.005	0.118	0.002
Stress threat (A)	0.425	0.307	0.043	0.427	7.097	0.000
Stress challenge (B)	0.454	−0.082	0.029	−0.122	−2.817	0.000
Stress threat (B)	0.464	0.020	0.043	0.028	0.461	0.019
Emotional regulation (B)	0.518	0.186	0.050	0.208	3.749	0.000
Emotional regulation (A)	0.537	0.175	0.050	0.196	3.478	0.001
**Anxiety after confinement**
Constant		−0.73	0.17		−0.4.27	
Public (B)	0.05	0.04	0.03	0.07	1.66	0.000
Stress threat (A)	0.43	0.30	0.04	0.42	6.84	0.000
Stress challenge (B)	0.44	−0.05	0.03	−0.07	−1.70	0.036
Stress threat (B)	0.45	0.02	0.04	0.03	0.52	0.023
Emotional regulation (B)	0.50	0.17	0.05	0.19	3.50	0.000
Emotional regulation (A)	0.51	0.14	0.05	0.15	2.78	0.007
Perspective taking (A)	0.52	0.10	0.04	0.09	2.12	0.035
**Stress after confinement**
Constant		−0.60	0.17		−3.44	
Public (B)	0.03	0.00	0.02	0.00	0.11	0.001
Emotional (A)	0.05	−0.00	0.03	−0.00	−0.23	0.013
Stress threat (A)	0.53	0.36	0.03	0.48	10.92	0.000
Emotional regulation (A)	0.60	0.21	0.04	0.23	4.64	0.000
Emotional regulation (B)	0.62	0.18	0.04	0.19	4.20	0.000
Perspective taking (A)	0.63	0.10	0.04	0.09	2.44	0.015

*B, before confinement; A, after confinement*.

Thirdly, to answer the third objective, repeated measures analyses were carried out according to country, Ecuador and Spain, as well as multiple linear regressions analysis. The results show that there were no significant differences in the analyzed variables according to the country before and after confinement (depression: *F* = 0.84, *p* = 0.35; anxiety: *F* = 0.01, *p* = 0.91; stress: *F* = 0.39, *p* = 0.52; public: *F* = 2.35, *p* = 0.09; emotional: *F* = 2.74, *p* = 0.06; altruistic: *F* = 1.54, *p* = 0.21; dire: *F* = 1.72, *p* = 0.18; compliant: *F* = 1.32, *p* = 0.26; anonymous: *F* = 0.37, *p* = 0.68; stress challenge: *F* = 0.04, *p* = 0.82; stress threat: *F* = 0.02, *p* = 0.86; secondary assessment: *F* = 2.41, *p* = 0.12; emotional contagion: *F* = 0.65, *p* = 0.54; empathetic action: *F* = 1.70, *p* = 0.19; perspective taking: *F* = 1.62, *p* = 0.20; emotional regulation: *F* = 0.41, *p* = 0.52; and self-awareness: *F* = 31, *p* = 0.58).

We computed six multiple linear regressions to gain insight into the predictive variables of depression, anxiety, and stress during-after confinement according to the country, from Ecuador and Spain.

The multiple linear regression analysis was performed separately for Ecuadorian and Spanish populations (Table 6). For depression, in the group from Ecuador, 61.8% (*R*^2^ = 0.61) of the variance is explained by the variables: public prosocial behavior (*B* = 0.08) and emotional regulation (*B* = 0.27) (before confinement), and stress threat (*B* = 0.41) and secondary assessment (*B* = −0.10) (after confinement). As regards to the group of Spanish population, 57.4% (*R*^2^ = 0.57) of the variance is explained by the variables: altruistic prosocial behavior (*B* = −0.21) and stress threat (*B* = 0.28) (after confinement), and stress challenge (*B* = −0.13) and emotional regulation (*B* = 0.19) (before confinement). The variable related to empathy, secondary assessment, altruistic prosocial behavior, and stress challenge had negative relations with depression. All other variables have positive relations.

**Table 6:** Multiple linear regression analysis by country, Ecuador and Spain.

	***R* squared**	***B***	**Standard error**	**Beta**	***t***	**Sig**.
**Depression after confinement**
**Ecuador**
Constant		−0.30	0.17		−1.71	
Public (B)	0.10	0.08	0.04	0.12	1.66	0.003
Stress threat (A)	0.52	0.41	0.05	0.58	7.34	0.000
Secondary assessment (A)	0.55	−0.10	0.04	−0.17	−2.39	0.014
Emotional regulation (B)	0.61	0.27	0.07	0.28	3.54	0.001
**Spain**
Constant		1.07	0.33		3.25	
Altruistic (A)	0.13	−0.21	0.06	−0.25	−0.3.34	0.001
Stress threat (A)	0.48	0.28	0.06	0.44	4.46	0.000
Stress challenge (B)	0.54	−0.13	0.05	−0.19	−0.2.52	0.003
Emotional regulation (B)	0.57	0.19	0.08	0.24	2.40	0.018
**Anxiety after confinement**
**Ecuador**
Constant		−0.71	0.28		1.06	
Public (B)	0.10	0.05	0.05	0.08	5.99	0.003
Stress threat (A)	0.50	0.37	0.06	0.49	3.29	0.000
Emotional regulation (B)	0.55	0.28	0.08	0.27	3.12	0.004
Emotional contagion (A)	0.59	0.24	0.07	0.24	−2.00	0.006
Self-Awareness (B)	0.61	−0.16	0.08	−0.14	1.06	0.049
**Spain**
Constant		−0.59	0.23		−2.535	
Dire (A)	0.05	0.22	0.07	0.35	3.199	0.037
Stress threat (A)	0.38	0.08	0.05	0.119	1.406	0.000
Emotional regulation (A)	0.45	0.27	0.08	0.352	3.207	0.002
**Stress after confinement**
**Ecuador**
Constant		−0.36	0.18		−2.04	
Public (B)	0.09	0.07	0.05	0.10	1.40	0.004
Stress threat (A)	0.51	0.42	0.06	0.54	6.83	0.000
Emotional regulation (B)	0.58	0.31	0.08	0.29	3.55	0.001
**Spain**
Constant		0.098	0.331		0.296	
Stress threat (A)	0.46	0.32	0.06	0.44	4.73	0.000
Emotional regulation (B)	0.53	0.39	0.08	0.44	4.54	0.001
Perspective taking (A)	0.57	−0.45	, 0.12	−0.35	−3.60	0.012
Perspective taking (B)	0.60	0.30	0.12	0.23	2.43	0.017

*B, before confinement; A, after confinement*.

Furthermore, for anxiety, in the Ecuadorian population, 61.7% (*R*^2^ = 0.61) of the variance is explained by the variables: public prosocial behavior (*B* = 0.05), emotional regulation (*B* = 0.28) and self- awareness (*B* = −0.16) (before confinement), and stress threat (*B* = 0.37) and emotional contagion (*B* = 0.24) (after confinement). In the Spanish population, 45.6% (*R*^2^ = 0.45) of the variance is explained by the variables: dire prosocial behavior (*B* = 0.22), stress threat (*B* = 0.08), and emotional regulation (*B* = 0.27), after confinement. Only the variable related to empathy, self-awareness, had a negative link with anxiety. All other variables had positive relations.

Finally, for stress, in the group of Ecuadorian population, 58.5% (*R*^2^ = 0.58) of the variance is explained by the variables: public prosocial behavior (*B* = 0.07) and emotional regulation (*B* = 0.31) (before confinement), and stress threat (*B* = 0.42) (after confinement). As regards to the group of Spanish population, 60.2% (*R*^2^ = 0.60) of the variance is explained by the variables emotional regulation (*B* = 0.39) and perspective taking (*B* = 0.30) (before confinement), and stress threat (*B* = 0.32) and perspective taking (*B* = −0.45) (after confinement). All these variables had positive relations with stress except for perspective taking after confinement, which was negatively related.

## Discussion

The present study intended to analyze the psychosocial effect of COVID-19, first by presenting a cross-sectional study in the Ecuadorian population and then through a comparative study between two samples of Ecuadorian and Spanish populations. Our study provides some important preliminary results regarding predictive relation that prosocial behavior, empathy, and the assessment of stress as a challenge has on depression, anxiety, and stress experienced by both the Ecuadorian and Spanish populations. It contributes to explain the variables and psychological processes that occur in adolescents as well as in the general population in the pandemic situation, especially the effects that the restrictions and control measures applied have had on the psychological adjustment to them.

As to the first objective of our study, the results have shown that the Ecuadorian adolescent population in general experienced significantly more depressive symptoms, anxiety, and stress after the confinement, as has also been shown in other recent studies [e.g., Orgilés et al., 2020; Breaux et al., 2021; Catagua-Meza and Escobar-Delgado, 2021; Echeverría Espinosa, 2021; Rogers et al., 2021; Sama et al., 2021]. As we have indicated before, the confinement and absence of schooling as a result of the pandemic has provoked social isolation and a breakup in interpersonal relationships, social, and physical interactions which, in the majority of the cases, has meant a negative effect on the mental health of children and young people at a worldwide level (Gatell-Carbó et al., 2021; Samji et al., 2021). There was a significant increase in behavioral and emotional problems, as well as sleep disorders, and a higher problematic use of the internet during and after confinement, which has contributed to this raise in depressive symptoms, anxiety, and stress (Chen et al., 2020; Moore et al., 2020; Pietrobelli et al., 2020; Xiang et al., 2020).

Regarding the second objective, our hypothesis was that prosocial behavior, together with a higher level of empathy and experiencing stress as a challenge (not as a threat), would act as a protector from depression symptoms, anxiety, and stress. Our results indicate that variables which help predict higher depression symptoms, higher anxiety, and stress in general experienced after confinement are: a higher public prosocial tendency; an empathetic tendency which implies a higher emotional regulation and, in the case of anxiety and stress, also, a higher perspective taking; and finally, a higher rate of assessing stress as a threat. Conversely, the variable stress assessed as a challenge contributes to predicting lesser depression symptoms and lesser anxiety while an emotional prosocial tendency contributes to predicting less stress in general experienced after confinement. Seeing these results, we can verify that our hypothesis has been fulfilled in part and in certain aspects.

First of all, and in relation to prosocial behavior, self-informed *emotional* prosocial tendency before confinement is what contributes to protecting from stress in general after confinement. This prosocial tendency refers to the prosocial action that the subject carries out in emotionally evoking situations, such as the health crisis, in which they can find themselves immersed (Carlo et al., 2003; Mestre et al., 2015). This result follows the same line as other research that, as indicated previously, has highlighted how situational and dispositional factors modulate prosocial behavior, explaining that the higher the ambiguity and gravity a specific situation presents the higher the probability exists of the appearance of helping behaviors (Batson and Powell, 2003; Galen, 2012; Hellman et al., 2021; Yue and Yang, 2021). When the motivation of the prosocial behavior is the emotionally evoking situation, we can affirm in base of our results, that this helping behavior protects from stress.

However, a tendency to behave prosocially with the intention to benefit others but in the presence of witnesses (*public*), meaning, when the prosocial motivation is the presence of others, predicts higher depression symptoms and higher anxiety. This result could be explained by a higher concern about the disapproval of others, by the prosocial motivation oriented to the desire to maintain a positive social image or to obtain the approval of others; it could also be explained by a motivation oriented to oneself, to self-satisfaction in front of others (Carlo and Randall, 2002; Eberly-Lewis and Coetzee, 2015; Davis et al., 2016; Alarcón and Forbes, 2017). They are prosocial behaviors, but they are motivated in a more selfish way (Davis et al., 2016).

Second of all, in our study, a higher empathetic emotional regulation and perspective taking perceived both before and after confinement has provoked a higher reporting of depressive symptoms, anxiety, and stress after confinement. These results are consistent with other studies (Schreiter et al., 2013; Tully et al., 2016; Calandri et al., 2019; van de Groep et al., 2020) in which the possible role of high levels of empathy in internalizing problems is analyzed, finding that high empathy could be a risk factor of depression. As in other studies, in our study it is not established that the lack or reduction of empathy is related to depression and anxiety (Llorca et al., 2014, 2017) but rather the opposite. In those studies, even though empathy does not appear directly related to depression, it is indirectly related to it through prosocial behavior. This could be due to empathy needing the modulator role of other variables, like the parenting styles of the father or mother, which contribute to channel and mediate between adequate levels of empathy and depression, anxiety, and stress (Mathews et al., 2016; Llorca et al., 2017). It can also be explained by the close relationship existing between empathy and depression. As indicated by some studies, the empathetic reaction to the distress of others, experienced during the situation of the pandemic, can result in personal distress. This can raise the risk of internalizing problems like depression (Tone and Tully, 2014; Yan et al., 2021). In any case, the results in this line are inconsistent in general.

Third of all, our hypothesis in regards to the variable of stress assessed as a threat and as a challenge is fulfilled. In this sense, when the subject assesses the stress perceived as a threat in situations prior to confinement, after it, the subject experiences higher levels of depression, anxiety, and stress in general. Conversely, when it is assessed as a challenge, the subject experiences less depression after confinement. These results follow the lines of those studies which show that the assessment of challenge works as a kind of efficient coping mechanism which allows the subject a higher level of wellbeing (Ramírez et al., 2008; Samper, 2014; Szkody and McKinney, 2020), experiencing fewer depressive symptoms.

In regards to the final objective, the analyses carried out show that in both countries the scores in depression, anxiety, and stress are higher after confinement, as was expected following the more recent related literature (Orgilés et al., 2020; Breaux et al., 2021; Catagua-Meza and Escobar-Delgado, 2021; Echeverría Espinosa, 2021; Rogers et al., 2021). However, there are differences in variables that help predict depression symptoms, anxiety, and stress.

First, in relation to the predictor effect of the self-informed prosocial behavior before confinement, results show that in Ecuador, the *public* prosocial tendency, meaning, the prosocial behavior carried out in the presence of others, is what predicts depression symptoms, as well as anxiety and stress experienced after confinement. As we have indicated previously, this kind of prosocial behavior looks for or needs for its execution a public recognition, thus distancing itself from the altruistic concept of prosociality. They are prosocial behaviors motivated in a more selfish way (Davis et al., 2016). In addition, this need for public approval has generated tension and anxiety which has manifested in internalized problems after the confinement period. Conversely, in Spain it has been the *altruistic* prosocial tendency which predicts fewer depression symptoms together with the *emergency* prosocial tendency, which predicts higher anxiety. The altruistic prosocial behaviors, as opposed to the public and emergency ones, are helping behaviors that are carried out with little or no expectation of reward for oneself (Carlo and Randall, 2002). They are mainly oriented to benefit others and they are motivated selflessly. These results are consistent with those found in other studies (e.g., Wilson and Musick, 1999; Chen et al., 2000; Davis et al., 2016) in which taking part in helping behaviors, in particular the altruistic kind, can induce a positive state of mind in whoever carries them out (Gueguen and De Gail, 2003), which could reduce negative emotional states like depression symptoms. In fact, it can help as a protective factor against depression symptoms.

Our results show, therefore, the differential predictive effects both prosocial tendencies have among both populations (Ecuadorian and Spanish) and suggest that those who help selflessly can obtain more benefits than those who help to benefit themselves.

Second, in relation to the predictor effect of self-informed empathy before confinement, the results have shown that there are no differences between Ecuador and Spain in the prediction of depression being a greater emotional regulation, which predicts, to a greater extent, higher depression symptoms. With regards to anxiety, empathy has more weight as a predictor in Ecuador. The dimensions of empathy which predict a higher anxiety has been a higher emotional regulation together with a higher emotional contagion, which allows to respond emotionally due to recognizing and understanding the emotional state of the other person, and a lesser self-awareness, defined as the ability to identify what the subject felt in the moment of affective excitement and at the same time evoking thoughts and feelings of others (Lamm et al., 2007; Richaud et al., 2017). In any case, and despite the fact that there have been more dimensions that evaluate empathy as predictors of anxiety in the population of Ecuador, the results of both populations indicate that high levels of empathy predict higher anxiety. These results can be explained due to the harmful effects of the pandemic, in particular, of the confinement, as other studies show (e.g., van de Groep et al., 2020), in which it was confirmed that the confinement had provoked a reduction of empathetic concern but a raise in perspective taking. In these studies, the harmful effects of the first weeks of confinement on empathetic response and on the opportunities for prosocial actions are shown, which are important predictors of a healthy socioemotional development. Other studies have shown that high levels of empathy in crisis situations are related to a higher level of support among the members of the family unit, which is generated especially in those who do more work or assistance (Siedlecki et al., 2014; Quílez-Robres et al., 2021) and can in turn develop higher anxiety.

Finally, as to the predictor effect of self-informed stress assessed as a threat or as a challenge before confinement by both populations, the results have shown that the stress assessed as a threat is a predictor variable in Ecuador as well as in Spain of depression, anxiety, and stress. But in Spain, stress assessed as a challenge aids in predicting depression. Therefore, in both populations, stress assessed as a threat fosters depressions symptoms, while in the Spanish population, stress assessed as a challenge protects against them. That is, this situation of health crisis, which prolongs the experience of stress, can involve anxiety, depression, and the inability to manage traumatic and negative emotions as we have stated. Furthermore, the constant fear of infection affects daily life and leads to social isolation, modifying human relations (Saladino et al., 2020).

This study has some limitations. The first limitation is that it was based on subjects' self-reported data. In future studies, it could be interesting to use alternative information sources to provide data on prosocial behavior, empathy, and the other variables. Another limitation is the type of sample that includes adolescents and young adults, which may have introduced some bias in the results. Finally, we have not included sociodemographic data, such as gender, age, and socioeconomic conditions, in the regressions analysis as covariates. These variables might be influencing the dependent variable. Future research might include them to evaluate their effect.

## Conclusion

The health actions against COVID-19 have brought about a rebirth of self-care, not only from the perspective of the individual who looks at their own survival, but also as an important member of society who needs to feel valued and accepted by it and in which society must show an interest (Villalobos, 2020). In this sense, sharing, helping, and having concern for others has been shown, in view of the results, as an important factor in this process. The population in general, but above all adolescents, have been deprived of a period of growth and personal development and of interpersonal relationships vital to this development and behavior and emotional self-regulation. It has been verified that taking part in altruistic prosocial behaviors leads to a better psychological adjustment. These results illustrate the potentially protective effects of the selfless helping behaviors against depression symptoms, anxiety, and stress.

The results bring to light, on the one hand, the similarities of the psychosocial effects that are being experienced independently of the country and, on the other hand, the differences in variables that can help explain these effects in the adolescent as well as the general Ecuadorian and Spanish populations. This can contribute to the creation of intervention plans which aim to soften and alleviate the effects produced by a situation like COVID-19, but also variables that should be taken into consideration in the prevention of depression and anxiety symptoms in the Ecuadorian and Spanish populations. Prosocial behaviors are not only indicators of morality and care for others, but are also an indicator of health and wellbeing (Carlo, 2014; Randall and Wenner, 2014; Davis et al., 2016). The development of prosociality with the related processes, empathy and emotional self-control when confronting situations that produce tension or before conflicts that require a solution from the subject, control or inhibit anxiety, aid in the development of an empathetic disposition, especially in the dimension of putting oneself in the place of another and to direct emotions to finding a solution, and are processes that should be taught and developed early to contribute to good emotional balance and psychological wellbeing.

## Data Availability Statement

The raw data supporting the conclusions of this article will be made available by the authors, without undue reservation.

## Ethics Statement

The studies involving human participants were reviewed and approved by the University of Azuay and University of Valencia. Written informed consent to participate in this study was provided by the participants' legal guardian/next of kin.

## Author Contributions

ÁC-O, PS-G, and EM-V made substantial contributions to the conception of the work. PS-G, EM-V, and AL-M selected the scales. ÁC-O was responsible for the data acquisition in Ecuador. PS-G, EM-V, AL-M, and AZ-A participated in the data collection in Spain. ÁC-O, PS-G, and VM-E wrote the manuscript, which all authors helped revise. AL-M and AZ-A revised references. All authors contributed to and approved the final manuscript.

## Conflict of Interest

The authors declare that the research was conducted in the absence of any commercial or financial relationships that could be construed as a potential conflict of interest.

## Publisher's Note

All claims expressed in this article are solely those of the authors and do not necessarily represent those of their affiliated organizations, or those of the publisher, the editors and the reviewers. Any product that may be evaluated in this article, or claim that may be made by its manufacturer, is not guaranteed or endorsed by the publisher.
